# Serum 25-hydroxyvitamin D levels in patients with cutaneous lupus erythematosus in a Mediterranean region

**DOI:** 10.1177/0961203309360807

**Published:** 2010

**Authors:** E Cutillas-Marco, MM Morales-Suárez-Varela, A Marquina-Vila, WB Grant

**Affiliations:** ^1^Department of Dermatology, University Hospital Dr Peset, Valencia, Spain; ^2^Unit of Public Health and Environmental Care, Department of Preventive Medicine, University of Valencia, Valencia, Spain; ^3^Research group CIBER CB06/02/0045 CIBER Actions-Epidemiology and Public Health; ^4^Research Foundation, University Hospital Dr Peset, Valencia, Spain; and ^5^Sunlight, Nutrition, and Health Research Center (SUNARC), San Francisco, California, USA

**Keywords:** Cutaneous Lupus, Subacute Lupus Erythematosus, Systemic Lupus Erythematosus, Vitamin D

## Abstract

Low vitamin D levels have been found in patients with autoimmune diseases, including type I diabetes, rheumatoid arthritis, multiple sclerosis and systemic lupus erythematosus. The main source of vitamin D is exposure to sunlight, but the same solar radiation is known to exacerbate lupus erythematosus. We investigated the prevalence of vitamin D insufficiency in patients with cutaneous lupus erythematosus (CLE). We designed a cross-sectional study including 55 patients with CLE to measure their serum 25-hydroxyvitamin D (25(OH)D) by chemiluminescence immunoassay and compare it with a control group consisting of 37 healthy sex and age-matched subjects recruited from the patients’ relatives as well as healthcare workers. Correlations with clinical and demographic variables were determined. Approximately 95% of patients with CLE had less than 30 ng/ml of serum 25(OH)D, which is accepted as the lower limit for vitamin D adequacy. Mean serum vitamin D values were significantly lower than controls (*p *= 0.038) and were associated with higher levels of parathyroid hormone (*p *= 0.050). A history of CLE was a strong predictor of insufficiency of vitamin D (odds ratio 4.2; 95% confidence interval 1.0–17.4). The results suggest a role of CLE in the metabolism of the vitamin and provide guidance for future studies looking at a potential role for vitamin D in the prevention and treatment of CLE.

## Introduction

Vitamin D is synthesized in human skin exposed to UV radiation.^1^ A lesser amount is obtained from food, although dietary sources supply less than 20% of the body’s requirement.^2^ Vitamin D requires a first hydroxylation in the liver to 25-hydroxyvitamin D (25(OH)D), which is used to determine vitamin D status, and then another hydroxylation in the kidney to its active form, 1-25-dihydroxyvitamin D3 (1,25(OH)2D). Definition of vitamin D insufficiency has changed over the past few years from less than 10 ng/ml to 40 ng/ml.^3^ Presumably, optimal levels will increase to 60 ng/ml as new functions are attributed to vitamin D.^4^ Concentrations of vitamin D are influenced by several factors such as diet, latitude, season, time spent outdoors, skin pigmentation, clothing, tanning habits and supplementation.^1^

Vitamin D has progressively become recognized as a pluripotent regulator of biological functions beyond its classical effects on bone and calcium homeostasis. The discovery that most tissues and cells in the body have a vitamin D receptor and the enzymatic machinery to activate 25(OH)D has improved our understanding of other non-classical effects of this vitamin.^1^ Of great interest is the role it can play in decreasing the risk of common cancers such as breast, colorectal and prostate cancer.^5^ Furthermore, evidence from large prospective studies in patients with rheumatoid arthritis,^6^ multiple sclerosis^7^ and type 1 diabetes^8^ suggest an important role for vitamin D as a modifiable environmental factor in autoimmune diseases. A high prevalence of vitamin D insufficiency and deficiency has been seen in patients with systemic lupus erythematosus (SLE).^9^^–^^13^. The link between vitamin D deficiency and CLE has not been as widely studied: previous studies found a high prevalence of vitamin D deficiency in patients with CLE, although they included patients with extracutaneous manifestations of lupus.^14^^,^^15^ Here, we test the hypothesis that inadequate levels of vitamin D are commonly found in patients with exclusive cutaneous manifestations of lupus erythematosus. Further, we will try to identify the clinical and analytical variables associated with vitamin D insufficiency as well as the potential relationship with severity of the disease.

## Materials and methods

### Patients

A cross-sectional study included patients with exclusively specific cutaneous manifestations of lupus erythematosus, whether chronic or subacute, attending a dermatology outpatient clinic in a public university hospital from May to October 2008. Patients with acute CLE were excluded owing to the strong association with SLE. Both patients and controls lived in Valencia, a city in southern Europe at 39° north 0° west, 11 m above sea level, with 65% average summer relative humidity, an annual precipitation rate of 454 mm, and many sunny days all year round. Only patients with more than a 1-year history of CLE, according to clinical, analytical and histological findings,^16^ were enrolled in the study. Patients who fulfilled at least four of the ACR criteria for the classification of SLE and those with extracutaneous manifestations of the disease were excluded from the study. CLE patients were compared with a control group consisting of 37 healthy sex and age-matched subjects (in two year bands) who were recruited from patients’ relatives as well as healthcare workers.

To participate in the study, patients and controls had to sign an informed consent, according to the Declaration of Helsinki. The local institutional review board approved the study protocol (study code CEIC 20/09).

### Methods

At enrolment, all participants were asked to complete a questionnaire covering age, gender, menopause, Fitzpatrick skin phototype, weight, height, photosensitivity, use of sunscreen, usual daily sun exposure including work activities, if currently a smoker, and number of cigarettes per day. Details of previous or current ongoing diseases, duration of lupus, type of cutaneous lupus, drugs and calcium and vitamin D supplements were obtained from their medical history. The daily average intake of vitamin D was estimated by measuring the amount of vitamin D-rich food eaten in a normal week. Assessment of CLE activity was based on the maximum number of exacerbations and days with active cutaneous lesions during the previous year, according to their medical history and interview.

Within a month of the inclusion of patients blood tests were performed: 25(OH)D, calcium, phosphorus, complement, antinuclear antibodies and intact parathyroid hormone (PTH). PTH and 25(OH)D were determined by chemiluminescence immunoassay. According to current recommendations,^9^^,^^10^^,^^17^ serum 25(OH)D levels <30 ng/ml and <10 ng/ml were defined as vitamin D insufficiency and deficiency, respectively.

### Statistical analysis

Age and levels of vitamin D in patients and controls were compared. All continuous variables, age and vitamin D were evaluated for normality of distribution, and only vitamin D in patients showed a skewed distribution. The data were expressed as mean ± standard deviation (SD) and were analysed by analysis of variance (ANOVA) test. A χ^2^ test was conducted to evaluate differences in percentage in the other dichotomic or categorical variables. Pearson’s correlation coefficients were calculated to describe the bivariate correlation among variables. Logistic regression models and odds ratio (OR) were also performed with deficiency of vitamin D, using a cut-off of 23.8 (mean of control group) as the outcome variable. For statistical inference, a bilateral *p*-value <0.05 was considered statistically significant. All analyses were calculated using the Statistical Package for Social Science (version 15, Chicago, Illinois, USA) for Windows.

## Results

### Demographic and lupus-related variables

Fifty-five patients were included in the study: the patients’ profile is summarized in Table 1. Most patients had CLE (80%), with a mean duration of 8.2 years since diagnosis was made. Photosensitivity was present in 69% of men and 59% of women. Sixty-seven per cent of patients presented at least one outbreak of cutaneous lesions during the previous year. The average number of exacerbations was 2.7 ± 3.7 and the mean period with active lesions was 108 ± 14.2 days. Eight patients (14%) were taking hydroxychloroquine and three women were receiving treatment with vitamin D+calcium supplements. None of the patients were receiving treatment with immunosuppressive drugs. The average calculated daily intake of vitamin D was 5.4 ± 2.2 µg. Forty-eight patients (87%) applied sunscreen to all uncovered skin, but only 30 (54%) used it every day assuring optimal protection during the middle hours of the day. Most of these patients used 50 or higher sun protection factor. UVexposure was less than 30 min/day in 20% of patients, whereas another 20% spent more than 2 h/day outside.

**Table 1:** Patients’ profile

Gender		Males 13 (23.6%)	Females 42 (76.4%)	p-value
Age (years)				
	Median	49.7	46.9	
	Standard deviation	11.8	13.9	0.52
	Range: minimum–maximum	35–77	23–84	
	over 50 years	7 (53.8%)	17 (40.5%)	
	over 60 years	2 (15.4%)	7 (16.7%)	0.39
				0.74
Skin type				
	Type II	1 (7.7%)	17 (40.5%)	0.06
	Type III	9 (69.2%)	13 (31.0%)	0.01
	Type IV	3 (23.1%)	12 (28.6%)	0.97
Body mass index				
	<24.9	6 (46.2%)	24 (57.1%)	0.48
	25.0–29.0	6 (46.2%)	9 (21.4%)	0.16
	>30.0	1 (7.7%)	9 (21.4%)	0.47
Cigarette smoking				
	Non-smokers	3 (23.1%)	16 (38.1%)	0.50
	Smokers	10 (76.9%)	26 (61.9%)	0.50
	Number of cigarettes a day (mean ± standard deviation)	15.5 ± 12.5	10.5 ± 11.3	0.17
Chronic renal disease		0 (0.0%)	1(1.8 %)	–
Chronic hepatic disease		2 (3.6%)	0 (0.0%)	–
Chronic cardiac disease		0 (0.0%)	1 (1.8%)	–
Menopause			16 (38.2%)	–
Duration of menopause				
	<10	–	8	–
	>10	–	8	–
Daily intake of vitamin D				
	<3.9	6 (46.2%)	9 (21.4%)	0.16
	3.9–6.7	5 (38.5%)	22 (52.4%)	0.38
	>6.7	2 (15.4%)	11 (26.2%)	0.66
Vitamin D levels (mean ± standard deviation)		19.67 ± 7.70	20.17 ± 9.36	0.67
Vitamin D supplements		–	3 (7.1%)	–

### Prevalence of vitamin D deficiency

**Figure 1:**
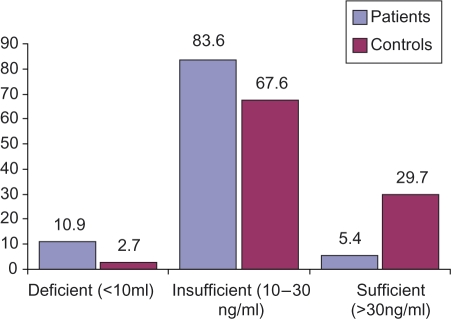
Prevalence of 25-hydroxyvitamin D deficiency and insufficiency in patients and controls. Serum levels of vitamin D of less than 30 ng/ml were found in 94.5% of patients, which is the range consistent with insufficiency, and includes six patients (10.9%) with less than 10 ng/ml. 25-hydroxyvitamin D levels in patients were significantly lower than controls (*p *= 0.03).

The overall mean (SD) 25(OH)D level was 20.0 ± 8.9 ng/ml in patients and 23.8 ± 7.5 ng/ml in controls. Differences between vitamin D levels in patients and controls were statistically significant and remained so after adjustment for age (*p *= 0.03). CLE was the strongest predictor of insufficiency of vitamin D (OR 4.2; 95% confidence interval (CI) 1.0–17.4). 25(OH)D levels decreased in older cases (*r *= −0.21), but this did not reach statistical significance (*p *= 0.12). However, this trend was not seen in controls (*r *= 0.13). Prevalence of vitamin D insufficiency and deficiency in cases and controls are shown in Figure 1. Higher levels of PTH were associated with lower serum levels of 25(OH)D (*r *= −0.27; *p *= 0.05), but no statistical significance was seen in controls.

We looked at other disease characteristics in association with 25(OH)D inadequacy but no other characteristic was found to be statistically significant in patients with a history of more than 10 years of lupus (*p *= 0.59), fair skin (*p *= 0.77), smoking (*p *= 0.56), photosensitivity (*p *= 0.39), detectable autoantibodies (*p *= 0.79), more than 180 days with active lesions within the last year (*p *= 0.38), and more than 2 h of outdoor exposure (*p *= 0.59). As shown in Table 2, treatment with hydroxychloroquine (OR 7.9; 95% CI 0.9–8.8) predicted vitamin D deficiency, whereas supplements of vitamin D were associated with higher vitamin D levels (*p *= 0.11).

**Table 2:** Risk of low vitamin D levels using 23.8 ng/ml (mean levels in controls) as the cut-off, in relation to different characteristics of cutaneous lupus erythematosus

		Odds ratio crude	95% confidence interval	
Age	<30	1.0 (Reference)		
	31–40	1.0	0.2	6.5
	41–50	1.5	0.2	8.9
	51–60	1.5	0.2	8.9
	>60	2.2	0.3	16.4
Menopause	No	1.0 (Reference)		
	Yes	3.3	1.1	9.8
Skin type	II	1.0 (Reference)		
	III	0.6	0.2	2.2
	IV	0.6	0.2	2.2
Body mass index	<24.9	1.0 (Reference)		
	25–29.9	0.9	0.2	2.5
	>30.0	0.8	0.3	3.9
Smoking	no	1.0 (Reference)		
	yes	0.9	0.3	3.0
Duration of disease	<4	1.0 (Reference)		
	4–9	2.6	0.6	11.3
	>9	1.3	0.3	5.3
Type of lupus	Subacute	1.0 (Reference)		
	Chronic	1.0	0.2	4.6
Daily intake of vitamin D	<3.9	1.0 (Reference)		
	3.9–6.7	1.2	0.3	4.6
	>6.7	0.8	0.2	3.7
Severity	<1	1.0 (Reference)		
	1–2	1.0	0.3–4.1	
	3–4	1.3	0.2–8.8	
	>4	3.2	0.5–19.0	
Antimalarials	No	1.0 (Reference)		
	Yes	7.9	0.9	69.8

## Discussion

We found significantly lower serum 25(OH)D levels among patients with CLE compared with matched controls and a high overall prevalence of vitamin D insufficiency (<30 ng/ml) and deficiency (<10 ng/ml). Insufficiency was seen in this population from Spain in the final spring, summer and early autumn months, the period of highest sun exposure, even in those patients with CLE who do not follow recommendations for sunscreen use or appropriate daily intake of vitamin D. We have found lower mean serum levels of vitamin D in Caucasian patients with SLE in South Carolina^10^ and Texas,^18^ probably due to the lower latitude, but similar levels to other studies performed in Spain.^9^ Mean serum levels of vitamin D in controls were also lower than controls in South Carolina.^10^ Our results are in line with other recent research on vitamin D levels in CLE that was performed in Germany.^14^ However, Cusack *et al*.^15^ observed a lower prevalence of vitamin D inadequacy in Ireland, at higher latitudes, which could be explained by the wider use of supplements of vitamin D (40% versus 5%).

Although the sample size is larger than other studies on vitamin D and CLE, lack of statistical significance in most variables could be attributed to the large number of factors that determines the capacity to synthesize vitamin D in the skin. This inadequacy in vitamin D levels in patients with CLE causes secondary hyperparathyroidism, which further exacerbates 25(OH)D deficiency by increasing its conversion to 1,25(OH)2D. Interestingly, we found an important role for the duration of the disease in lower 25(OH)D levels. The influence of age in levels of vitamin D in patients but not in controls may reflect the role of the disease in the metabolism of vitamin D.

Previous studies on CLE^14^ could not find a relationship with the severity of the disease based on indexes such as the European Consensus Lupus Activity Measurement (ECLAM), SLEDAI or SLICC Damaged Index (SDI), perhaps because these scales were developed and validated specifically for patients with SLE^19^ and not for CLE. In view of the lack of an internationally validated index to score CLE activity, we used a clinical disease-related parameter, and a trend toward lower vitamin D levels is seen in patients with more active disease. Some studies found a negative correlation between vitamin D levels and severity of SLE,^2^^,^^11^^,^^18^ while others^9^ only found an association between vitamin D deficiency and fatigue. Interestingly, in other autoimmune disorders such as undifferentiated connective tissue disease, the probability of developing dermatological symptoms correlated with vitamin D insufficiency.^17^

Our data disclosed two apparent contradictions: antimalarials seemed to be a predictor of lower 25(OH)D levels; hydroxychloroquine is known to inhibit the 1α-hydroxylation of 25(OH)D, thus decreasing the levels of 1,25(OH)2D.^9^ Our results may be due to the severity of the disease, as patients receiving treatment with hydroxychloroquine were those with active lesions in spite of photoprotection and topical therapy. The finding of lower 25(OH)D levels in patients with longer periods of active lesions and more exacerbations during a year would support this possibility. Nevertheless, the low absolute number of patients who took hydroxychloroquine represents an additional limitation on the interpretation of data related to this variable. Further research controlling for severity of the disease may clarify the influence of antimalarials on vitamin D levels in CLE patients. The other paradox was the finding of higher 25(OH)D levels in darker skin, although all our patients were Caucasians. It could be explained by the lack of photosensitivity in patients with Fitzpatrick skin type IV. Another confounding factor, time spent outdoors, could be behind this finding.

Although more studies are needed to understand the role of vitamin D as an immunomodulator, the existence of vitamin D receptors in T and B lymphocytes, macrophagues and dendritic cells is well known. Perhaps the most profound effects on the immune system are on dendritic cells,^10^ modulating their maturational state. Furthermore, 1,25(OH)2D down-regulates dendritic cell production of interleukin-12 and augments interleukin-10, consisting of a shift towards a Th2 response. B lymphocyte differentation is also interrupted when exposed in vitro to vitamin D. Although increasing levels of dietary vitamin D intake are not associated with a decreased risk of autoimmune diseases,^12^ administration of supplementary vitamin D to mouse models of SLE resulted in a loss of dermatological manifestations.^20^ Based on our findings and evidence in the literature, it is convenient to look for vitamin D deficiency in CLE, although further research is necessary to demonstrate the benefits of correcting vitamin D status in these patients.
